# Revealing dietary habits and intestinal microbiome composition of the Beijing swift (*Apus apus pekinensis*) through regurgitated pellets and fecal samples

**DOI:** 10.3389/fmicb.2025.1693396

**Published:** 2025-12-01

**Authors:** Bojun Wang, Yaqiong Zhang, Jie Li, Mengwei Wu, Yingliang Shi, Xiangnan Sun, Xin Xiao, Pan Zhang, Yang Shi, Yimeng Li, Hengjiu Tian

**Affiliations:** 1 Beijing Wildlife Rescue and Rehabilitation Center, Beijing, China; 2 Department of Life Sciences, Natural History Museum of China, Beijing, China

**Keywords:** *Apus apus pekinensis*, regurgitated pellets, fecal samples, dietary habits, intestinal microbiome

## Abstract

**Introduction:**

The Beijing swift, an important insectivorous bird, is a key protected wild animal in Beijing. Current research on this species primarily focuses on distribution surveys and population dynamics, while systematic studies on its diet and intestinal microbiome composition remain lacking, a knowledge gap that constrains in-depth understanding of its ecological adaptability.

**Methods:**

This study integrated DNA barcoding and high-throughput 16S rRNA gene sequencing to systematically analyze regurgitated pellets and fecal samples from ringed and rescued individuals, revealing the following findings.

**Results:**

The dietary composition primarily encompasses insects from five orders, Diptera, Coleoptera, Hemiptera, Hymenoptera and Lepidoptera, with significant differences observed between adults and nestlings. Dominant intestinal bacterial phyla included Proteobacteria, Firmicutes, Bacteroidota, and Actinobacteriota. Correlation network analysis indicated that Stenotrophomonas, Aminobacter, etc., exhibit extensive mutually promotive interactions with other bacteria, suggesting their potential roles as core functional bacterial communities in the intestine.

**Discussion:**

This research provides the first evidence of dietary differentiation patterns and intestinal microbial composition characteristics of Beijing swifts, providing key foundational data for assessing its survival and adaptation mechanisms. It is highly significant for developing scientific rescue strategies and conservation initiatives.

## 1 Introduction

The Beijing Swift (*Apus apus pekinensis*), belonging to the order Caprimulgiformes and family Apodidae, is a subspecies of the Common Swift (*Apus apus*) endemic to Beijing. This subspecies primarily breeds in northern China and is a summer migratory bird in Beijing. It undertakes long-distance transcontinental migration during spring and autumn, with its wintering grounds located in continental Africa (Akesson et al., 2012). The Beijing Swift has a strong flying ability and can complete activities such as hunting, collecting nesting materials, courtship, and mating while in flight. It basically does not land except during the breeding season (del Hoyo et al., 1999). The Beijing Swift is a key protected animal in Beijing and listed as Least Concern on the IUCN Red List. Current research on swifts primarily focuses on distribution and population surveys, as well as evolutionary and ecological biology. Limited existing studies indicate that Beijing swift primarily feeds on insects of Diptera, Hemiptera, and Lepidoptera orders (Wang, 1985; Kopij, 2000), including substantial quantities of mosquitoes, flies, and tabanids. Consequently, this subspecies serves as a significant biological control agent against pests and is recognized as an insectivorous bird of ecological value (Zheng, 1993). No studies to date have analyzed the dietary composition of Beijing swift through both regurgitated pellets and fecal samples, nor has any research elucidated its intestinal microbiota composition. This knowledge gap impedes a comprehensive understanding of the swift’ survival status in Beijing and hampers efforts to develop nutritional rehabilitation protocols and conduct health assessments for swift conservation.

As a crucial “microbial organ” within the animal body, the intestinal microbiota participates in host nutrient absorption, metabolic regulation, and immune responses. It plays critical roles in maintaining organismal health and facilitating adaptive evolution (De Vos et al., 2022; Zhang et al., 2025). As essential indicator group in ecosystems, birds have attracted increasing research attention regarding their intestinal microbial composition and function in recent years (Sun et al., 2022; Bodawatta et al., 2022). Birds’ complex life history traits, diverse diets, specialized physiological architecture, and long-distance migrations impose heightened selective pressures on their physiological processes, thereby driving intricate dynamics in intestine microbiota. The composition and diversity of avian intestinal microbiomes are modulated by species-specific factors, environmental conditions, life history stages, etc., (Somers et al., 2023; Li et al., 2025), with dietary exerting particularly profound influences on microbial composition (Youngblut et al., 2019).

This study employs DNA metabarcoding and 16S rRNA gene sequencing to analyze regurgitated pellets and fecal samples from Beijing swift banded at Kuoru Pavilion in the Summer Palace and the Beijing Wildlife Rescue and Rehabilitation Center. We hypothesized that significant differences exist not only in the diet composition detected between regurgitated pellets and fecal samples, but also in the intestinal microbiota composition of Beijing Swifts between healthy, wild and injured or rehabilitated states. This research aims to elucidate the dietary composition and intestinal microbiota structure of Beijing Swifts in Beijing area, providing scientific basis for the subsequent conservation of this bird.

## 2 Materials and methods

### 2.1 Sample collection

During Beijing swift banding operations at Kuoru Pavilion in the Summer Palace on 1 June 2024, 12 freshly regurgitated pellets and 15 freshly fecal samples were collected from different individuals. As adult Beijing Swifts carry food back in their mouths for their young, the food pellets regurgitated by some individuals during banding serve as a direct source of the nestlings’ food source, whereas fecal samples represent the dietary intake of adults. In addition, from May to August 2024, 15 fecal samples were obtained from newly rescued Beijing swifts at the Beijing Wildlife Rescue and Rehabilitation Center prior to artificial feeding. This yielded a total of 42 pellet and fecal specimens from both sites, with sample grouping details provided in Table 1. Samples were collected using the following protocol: Beijing Swifts carrying food boluses were captured in mist nets during their return to nesting sites. Regurgitated pellets expelled during net entanglement were collected with sterile surgical gloves, placed in 5 mL sterile centrifuge tubes, sealed, and labeled with corresponding information. During banding or rehabilitation procedures, fecal samples were collected immediately with sterile cotton swabs after defecation. The swab tips were aseptically severed into 1.5 mL sterile centrifuge tubes, sealed, and labeled with corresponding information. Both regurgitated pellets and fecal samples were stored in portable refrigerators before subsequent transfer to laboratory −80 °C freezers for preservation and further analysis.

**Table 1:** Sample group information.

Group	Sample number	Sample source
Regurgitated Pellets (Group A)	A1–A12	Ringed individuals from Kuoru Pavilion in the Summer Palace
Fecal samples (Group B)	B1–B15	Ringed individuals from Kuoru Pavilion in the Summer Palace
Fecal samples (Group C)	C1–C15	Rescued individuals at the Beijing Wildlife Rescue and Rehabilitation Center

### 2.2 DNA extraction and sequencing

For pellet samples, fine grinding with liquid nitrogen and a mortar is necessary to thoroughly pulverize the chitinous exoskeletons prior to DNA extraction. For fecal samples, the swab tip containing the sample is immersed in a buffer and vortexed to achieve a homogeneous dispersion. Large insoluble particles are then removed by low-speed centrifugation, and the supernatant is proceeded to the lysis step. The QIAamp Fast DNA Stool Mini Kit (QIAGEN, Hilden, Germany) and DNeasy PowerSoil Pro Kit was used in accordance with the manufacturer’s protocols for the extraction of fecal DNA and bolus DNA, respectively. Genomic DNA concentration and purity were measured using a Qubit fluorometer (Thermo Fisher Scientific, United States). Bacterial community composition was assessed by sequencing the V3-V4 region of 16S rRNA genes using PCR primers 338F (5′- ACTCC-TACGGGAGGCAGCA -3′) and 806R (5′- GGACTACHVGGGTWTCTAAT -3′), while the dietary composition in both regurgitated pellets and fecal samples was determined by sequencing the Cytochrome Oxidase I (COI) gene using primers F (5′- GGWACWGGWTGAACWGTWTAYCCYCC -3′) and R (5′- TAAACTTCAGGGTGACCAAARAAYCA -3′). The PCR system contained 2.5 ng of template DNA, 2 μL of dNTP, 5 μL of KOD FX Neo Buffer, 0.2 μL of KOD FX Neo, 0.3 μL each of primer pairs, add ddH₂O to a final volume of 10 μL. PCR conditions were initiated at 95 °C for 5 min, followed by 25 cycles of denaturation at 95 °C for 30 s, annealing at 50 °C for 30 s, and extension at 72 °C for 40 s, followed by a final elongation at 72 °C for 7 min, and then hold at 4 °C. Quant-iT dsDNA HS kit was applied to do the PCR products quantification. Paired-end sequencing was conducted by Biomarker Technologies Co. Ltd. (Beijing, China) using the Illumina NovaSeq 6000 platform (Illumina Inc., San Diego, CA, USA).

### 2.3 Data processing and analysis

Using FLASH v1.2.7 software, paired-end reads from each sample were assembled via overlap, generating assembled sequences designated as Raw Tags. Raw Tags were filtered using Trimmomatic v0.33 to obtain high-quality Clean Tags. Chimeric sequences were identified and removed with UCHIME v4.2, yielding final Effective Tags. Tags were clustered into OTUs at 97% similarity threshold using UCLUST in QIIME. Representative OTU sequences were aligned against the Barcode of Life Data System (BOLD) database and SILVA v132 database, respectively, to assign taxonomic classifications. Alpha diversity indices (ACE and Shannon), which indicate microbial community richness and diversity, were calculated using QIIME2. Non-metric multidimensional scaling (NMDS) together with Analysis of Similarity (ANOSIM) was used to identify differences in bacterial community composition between the sampling sites. Co-occurrence network analysis was performed on bacterial genera (top 50 genera relative abundance, Spearman rank *r* > 0.8 or *r* < −0.8 were used) to predict the potential interactions between different genera. Interaction network metrics and plots were analyzed and visualized with Gephi v0.9.2 software. Values of taxa abundance for the dietary composition and intestinal microbiota are reported as mean ± SE. Student’s *t*-test and One-way ANOVA were used for data analysis. A *p*-value < 0.05 was considered statistically significant, while a *p*-value < 0.01 indicated highly statistical significance.

## 3 Results

### 3.1 Statistical analysis of sequencing data

For 16S rRNA genes (V3-V4) sequencing, a total of 2,908,051 raw reads were generated. After removing low-quality and chimeric sequences, 2,569,539 effective reads were obtained, ranging from 37,054 to 73,825 per sample. Cluster analysis yielded 642 Amplicon Sequence Variants (ASVs), which were taxonomically classified into 10 phyla, 25 classes, 35 orders, 62 families and 75 genera. The rarefaction curves (Supplementary Figure S1a), which reached a clear plateau, indicated that the observed species increased with sequencing depth and that the sequencing effort was sufficient for subsequent analysis.

COI gene sequencing produced 2,295,008 raw reads. Following quality filtering and chimera removal, a final set of 2,021,117 high-quality reads was obtained, with the count per sample ranging from 46,085 to 73,342. These reads were clustered into 20,839 ASVs, which were taxonomically classified into 47 phyla, 118 classes, 329 orders, 704 families and 1,799 genera. The rarefaction curves (Supplementary Figure S1b) attained a clear plateau, demonstrating that adequate sequencing depth was achieved.

### 3.2 Diet composition of Beijing swift

**Figure 1:**
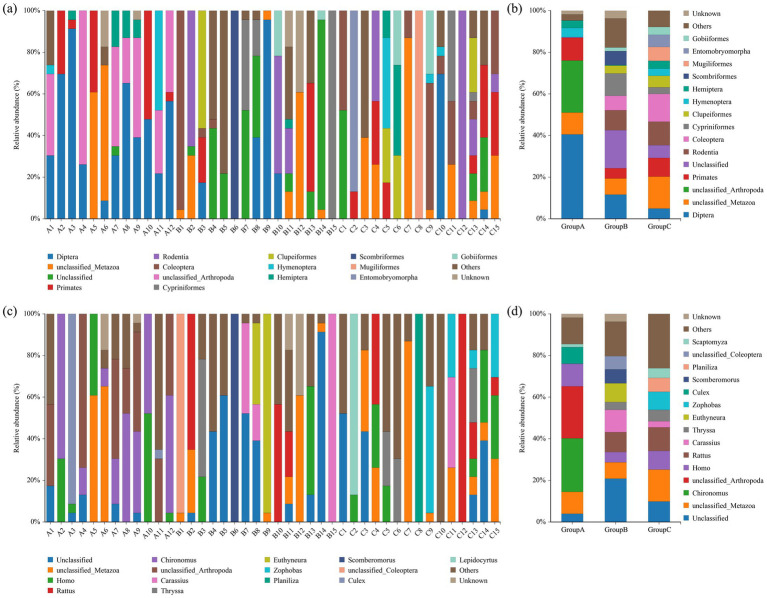
Bar chart of relative abundance. Relative abundance (%) of the top 15 abundant bacteria order **(a)** for individuals, **(b)** for groups and genera **(c)** for individuals, **(d)** for groups obtained from regurgitated pellets and fecal samples of Beijing swift. Others: Bacteria taxa with ≤1% abundance.

**Figure 2:**
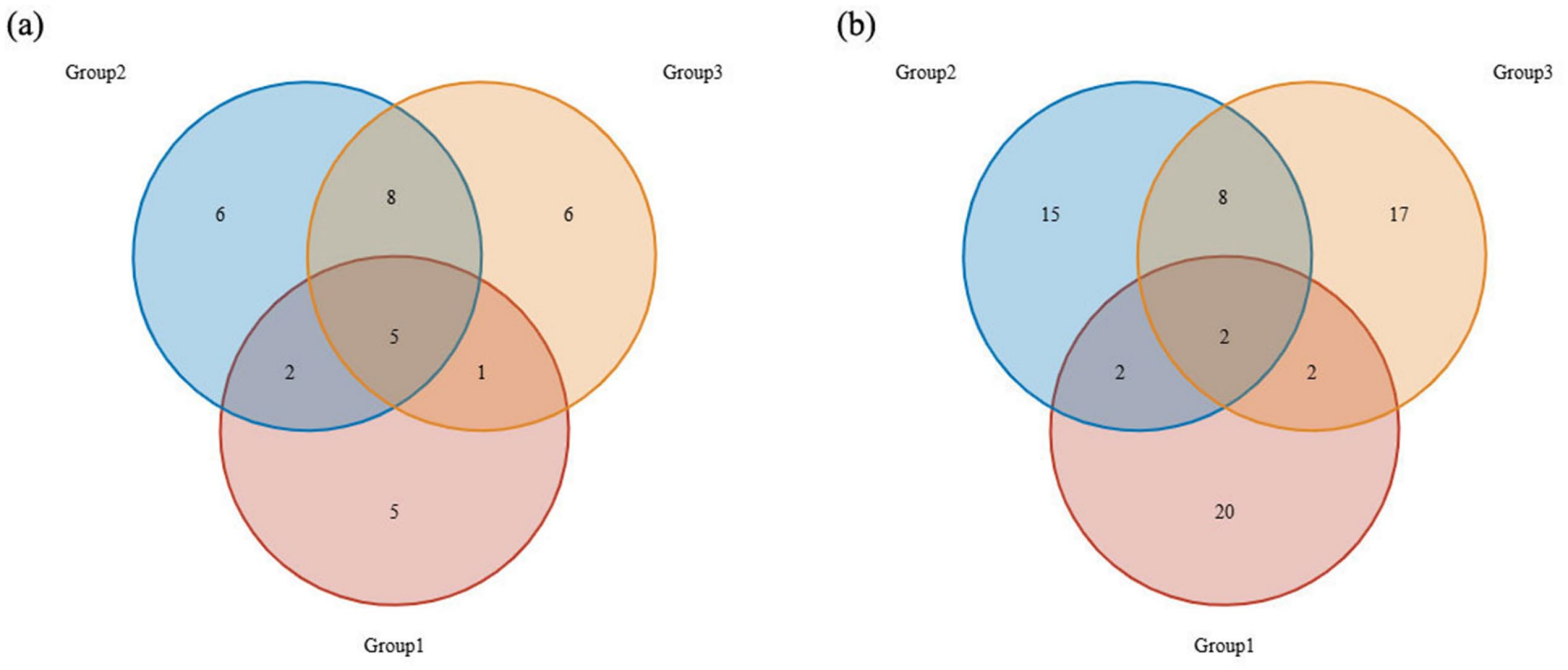
Venn diagram. The Venn diagrams show the numbers of **(a)** orders and **(b)** genera that were shared or not shared by all three groups, respectively.

**Figure 3:**
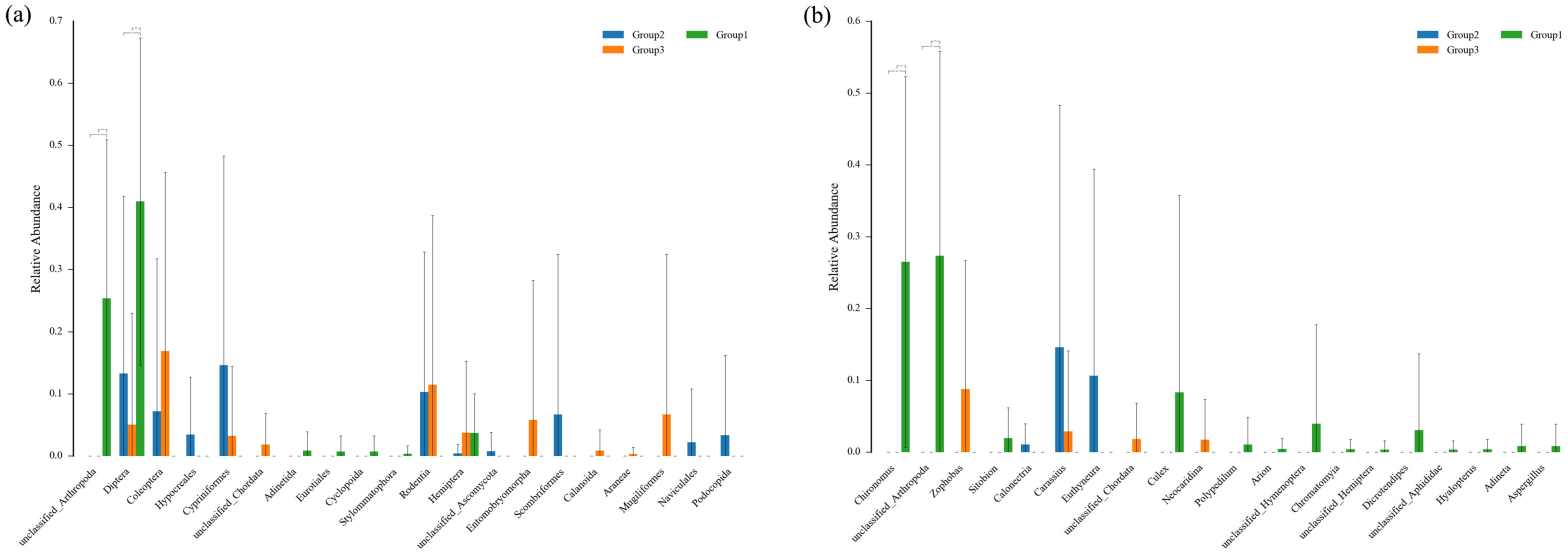
Bar plots illustrating differences in taxonomic abundance. Bar plots showing abundance differences at the **(a)** orders and **(b)** genera levels. The values represent the mean ± SE. *p < 0.05 reflects significant differences and **p < 0.01 reflects highly significant differences.

The top 15 most abundant orders in the dietary composition of the Beijing Swift are shown in Figures 1a,b. Overall, the diet of the Beijing Swift primarily consists of insects from five orders: Diptera, Coleoptera, Hemiptera, Hymenoptera, and Lepidoptera. In regurgitated pellets (food sources for nestlings), Diptera accounted for a relatively high proportion (41.33% ± 7.47%), followed by Hemiptera (3.24% ± 1.36%). In fecal samples, the abundance of Diptera was lower, the values are 10.53% ± 6.65 and 1.44% ± 1.12%, respectively, and were detected only in a small subset of individuals. Statistical analysis revealed that the abundance of Diptera was significantly higher in pellets than in fecal samples (*p* < 0.05). The abundance of Coleoptera was 0.25% ± 0.15% in the food bolus and 7.33% ± 6.35 and 12.61% ± 5.21% in feces, respectively; the abundance in feces was higher than that in the food bolus, but the difference was not statistically significant (*p* > 0.05). Figures 1c,d shows the top 15 most abundant food genera. *Chironomus* and *Culex* were only detected in regurgitated pellets, with relatively high abundances of 25.86% ± 7.23 and 8.02% ± 7.47% respectively, and were not detected in fecal samples. The relative abundance of *Chironomus* was significantly higher in pellets than in fecal samples (*p* < 0.05). *Zophobas* and *Scaptomyza* showed higher abundance in Beijing Swifts rescued by the Beijing Wildlife Rescue and Rehabilitation Center, but were not detected in regurgitated pellets or fecal samples from ringed birds at the Summer Palace. The detection of *Zophobas* genus in rescued individuals C9, C11, C13, and C15 is likely attributable to artificial feeding with barley pest prior to rehabilitation. Venn diagram reflects the similarities and differences in dietary among the three groups at the order (Figure 2a) and genus (Figure 2b) levels, respectively. The three groups shared 5 orders and 2 genera. Figure 3 shows the orders and genera with significant differences among the three groups.

### 3.3 Intestinal microbiome composition of the Beijing swift

**Figure 4:**
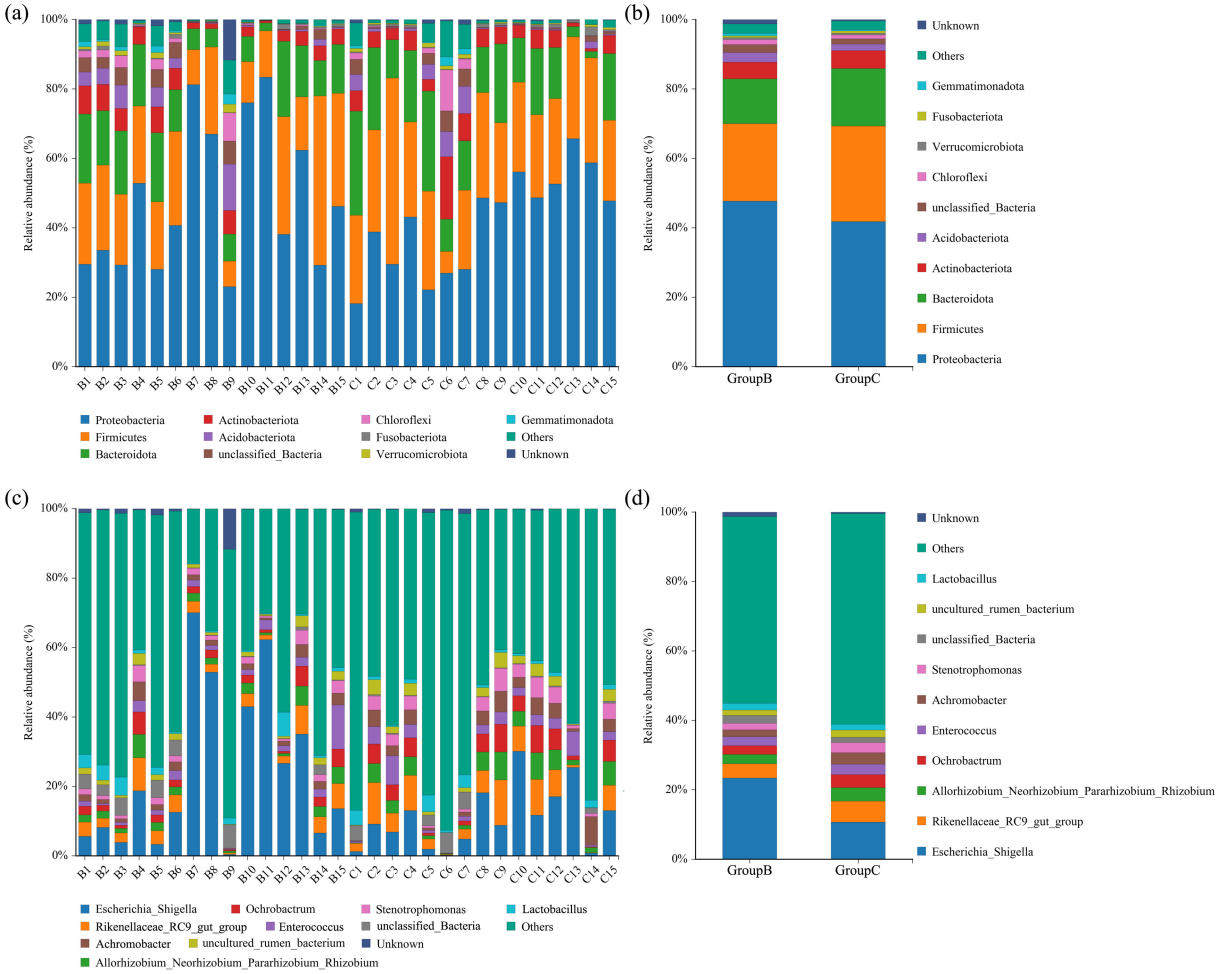
Bar chart of relative abundance. Relative abundance (%) of the top 10 abundant bacteria phyla **(a)** for individuals, **(b)** for groups and genera **(c)** for individuals, **(d)** for groups obtained from regurgitated pellets and fecal samples of Beijing swift. Others: Bacteria taxa with ≤1% abundance.

The top 10 most abundant bacterial phyla in the intestine of the Beijing Swift are shown in Figures 4a,b. The dominant phyla were Proteobacteria, Firmicutes, Bacteroidota, and Actinobacteriota. In fecal samples collected from the Summer Palace, the relative abundances of these dominant phyla were 48.04% ± 5.42, 22.33% ± 2.76, 12.87% ± 1.58, and 4.67% ± 0.62%, respectively. In fecal samples collected from the Rescue Center, the relative abundances were 42.15% ± 3.69, 26.91% ± 2.43, 16.28% ± 2.15, and 5.33% ± 1.01%, respectively. No significant differences in the relative abundance of these dominant bacterial phyla between the two sampling sites (*p* > 0.05). Figures 4c,d shows the top 10 most abundant bacteria genera. The dominant genera were *Escherichia_Shigella* and *Rikenellace-ae_RC9_gut_group*. In Summer Palace fecal samples, their relative abundances were 24.18% ± 5.96 and 4.08% ± 0.66%, respectively. In Rescue Center fecal samples, their relative abundances were 10.81% ± 2.32 and 5.93% ± 1.10%, respectively. The relative abundance of *Escherichia_Shigella* was significantly higher at the Summer Palace fecal samples than at the Rescue Center fecal samples (*p* < 0.05).

### 3.4 Differences in intestinal microbiota composition and diversity between the two sampling sites

**Figure 5:**
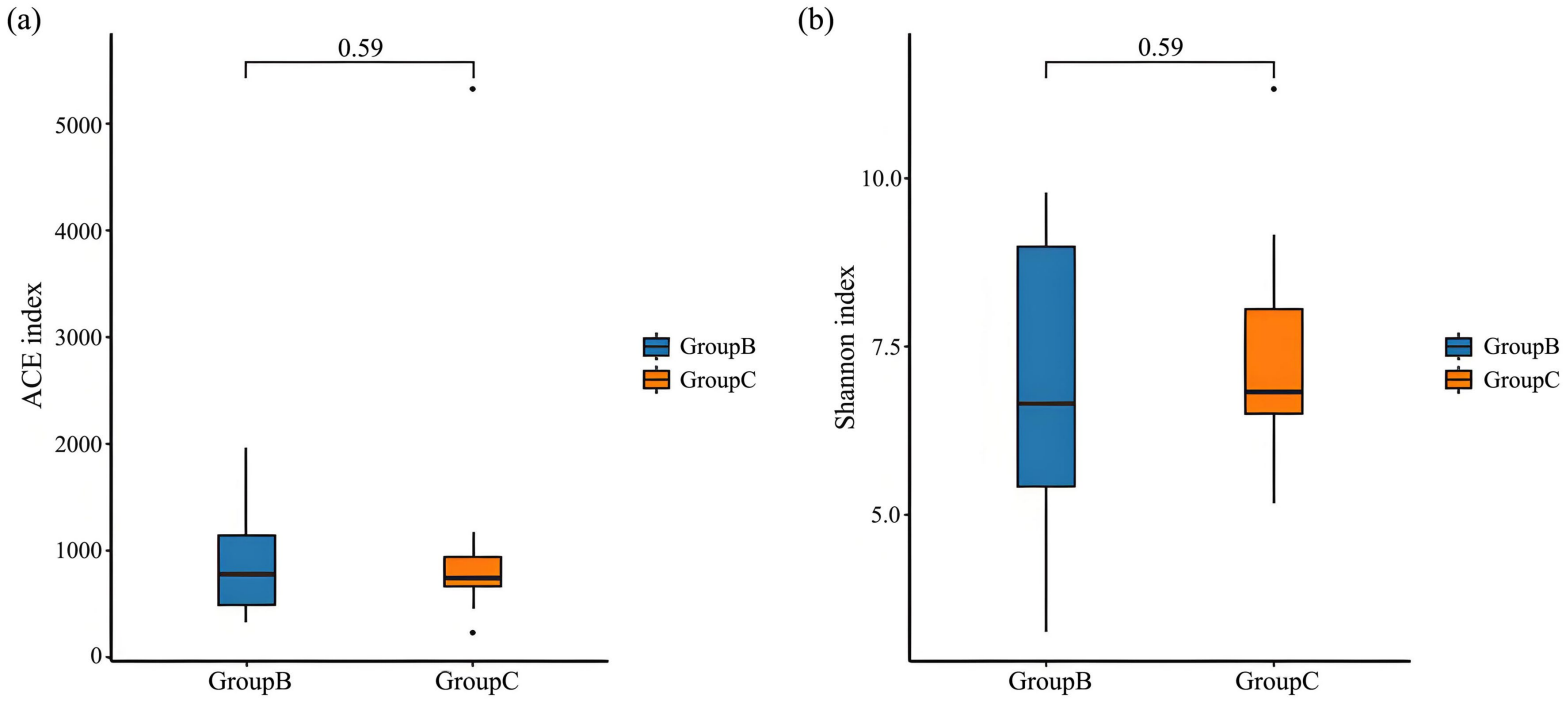
Box plot of alpha diversity indices. **(a)** ACE index and **(b)** Shannon index of intestinal microbiome of the Beijing swift. Boxes represent the interquartile range (25th–75th percentiles), and the horizontal line inside the box indicates the median. *p* > 0.05 reflects no significant differences.

**Figure 6:**
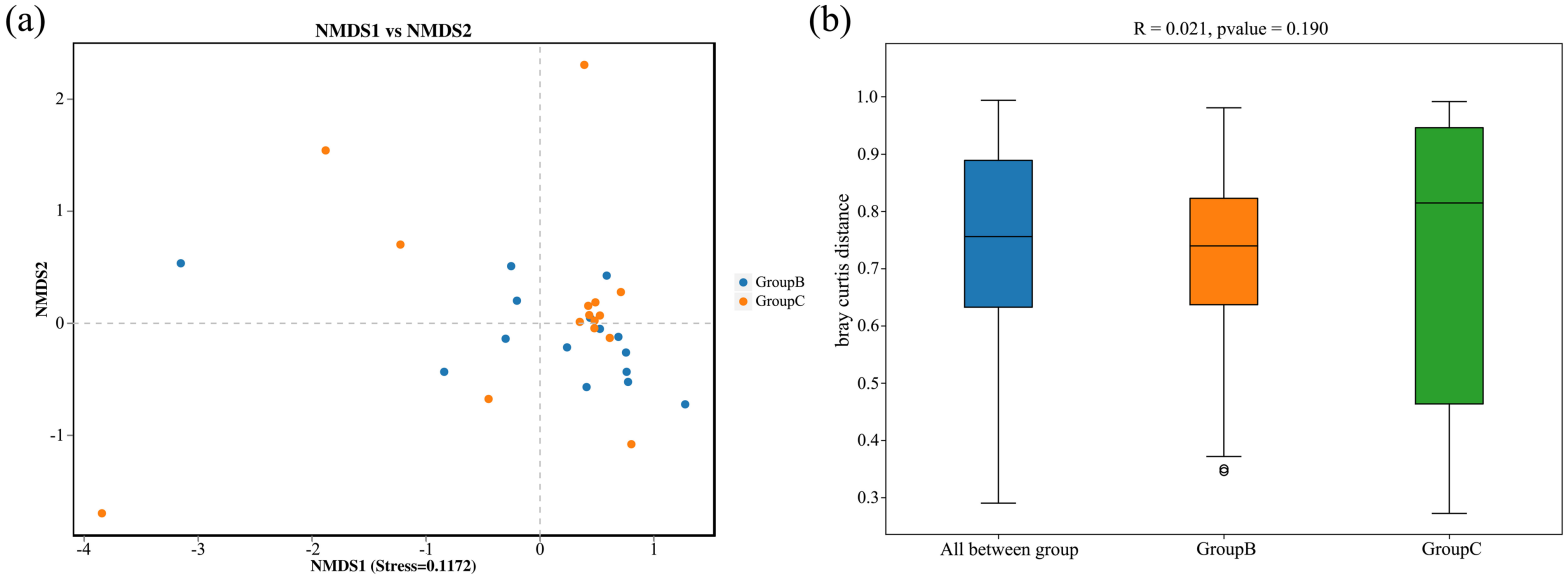
Beta diversity. **(a)** NMDS plots and **(b)** ANOSIM distance boxplot. NMDS plots were constructed using Bray–Curtis. The closer the spatial distance of the sample, the more similar the bacterial composition of the sample. Boxes represent the interquartile range (25th–75th percentiles), and the horizontal line inside the box indicates the median.

For the intestinal microbiota of Beijing Swifts from the Summer Palace and the Rescue Center sampling sites, at the alpha diversity level, ACE index values were 882.01 ± 126.58 and 1064.17 ± 310.33 respectively, and Shannon index values were 6.80 ± 0.57 and 7.35 ± 0.42 respectively, indicating no significant differences in richness or diversity between the two sites (Figure 5). At the beta diversity level, NMDS analysis revealed close clustering of most samples from both sites, excluding a few outliers. Combined with ANOSIM results (R = 0.021, *p* = 0.19), these findings demonstrate no significant difference in intestinal microbiota composition between the two sampling sites (Figure 6).

### 3.5 Correlation network of the top 50 most abundant bacterial genera

**Figure 7:**
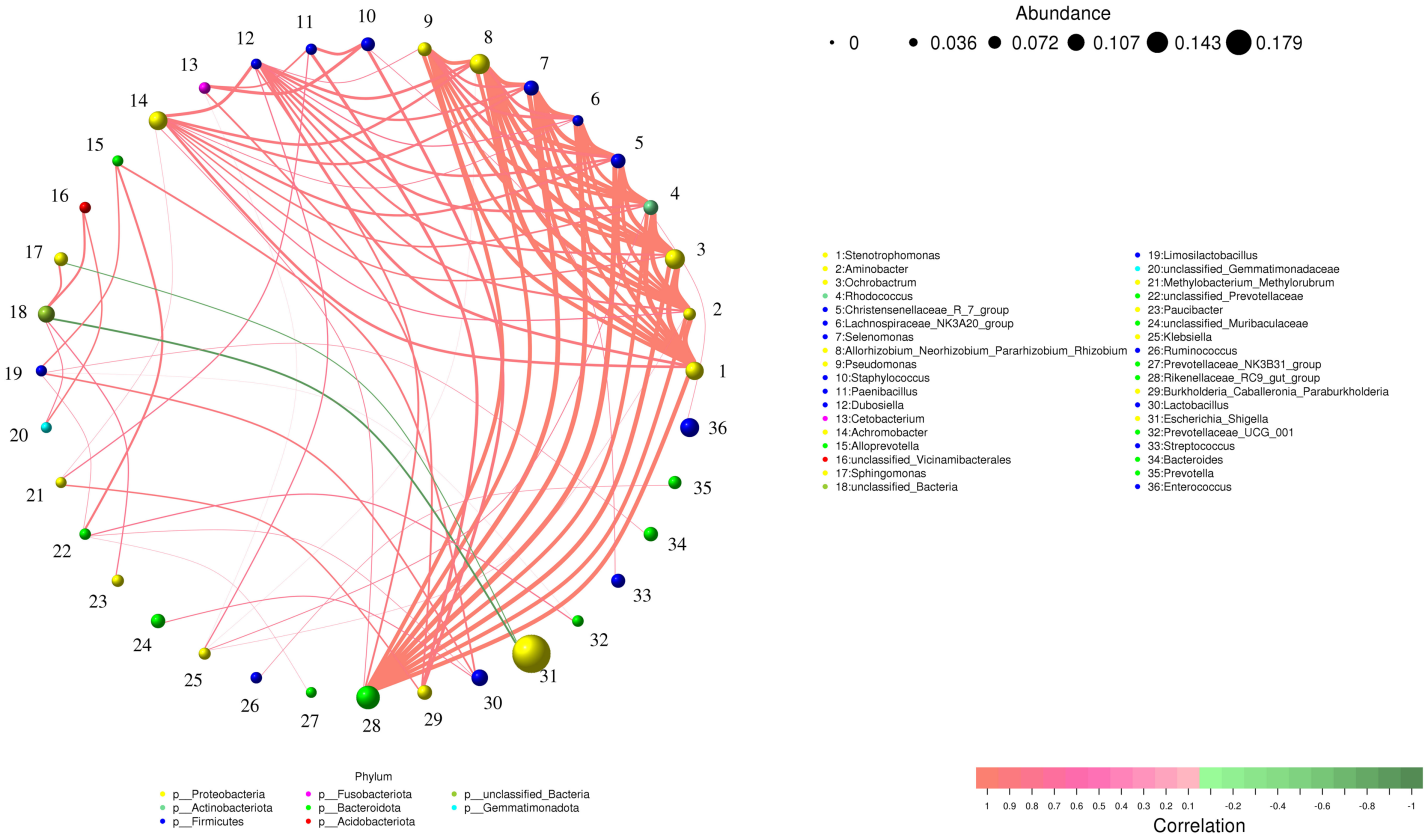
Correlation Network analysis of the top 50 bacterial genera in the intestine of Beijing swift. The network is displayed by nodes (genera) and edges (relationship between the nodes). A connection stands for a strong (Spearman rank *r* > 0.8 or *r* < −0.8) and significant (*p* < 0.05) correlation between the genera. The size of a node depends on the number of connections. The same node color indicates genera belong to the same phylum. A red edge indicates a positive interaction between two individual nodes, while a green edge indicates a negative interaction.

Figure 7 displays the correlation network of the top 50 most abundant bacterial genera. It reveals that almost all intergenus relationships are positive correlations, except for the negative correlations observed between *Escherichia_Shigella* and two other genera (*unclassified_Bacteria* and *Sphingomonas*). Notably, genera such as *Stenotrophomonas*, *Aminobacter*, *Ochrobactrum*, *Rhodococcus*, exhibit extensive interactions with other genera, suggesting they may serve as key taxa in the intestine of Beijing Swifts, potentially engaging in mutually promotive relationships with other bacteria. Additionally, *Prevotella* shows only one interaction, a positive correlation with *Rumi-nococcus*.

## 4 Discussion

This study through the collection of regurgitated pellets and fecal samples from Beijing Swifts banded at the Kuoru Pavilion in the Summer Palace and those rescued by the Beijing Wildlife Rescue and Rehabilitation Center, revealed for the first time the dietary composition and intestinal microbiome structure of this insectivorous bird.

Significant divergences were observed in both insect taxa and their abundances identified in regurgitated pellets versus feces, demonstrating the foraging strategy of adult birds during the nesting period. In regurgitated pellets, the abundance of Diptera was significantly higher than that in the two fecal sample groups (*p* < 0.05). While, the abundance of Coleoptera detected in feces was higher than that in boluses but no significant difference was observed (*p* > 0.05). *Chironomus* and *Culex* were exclusively detected at relatively high abundances in regurgitated pellets but were not detected in fecal samples. There are two possible reasons for this difference: first, the hard bodies of Coleoptera and their high chitin content make them difficult to digest. In contrast, Diptera have thin body walls and are easier to digest. Therefore, it is speculated that adult birds feed more Diptera to their young to ensure higher digestibility and better nutrient absorption. During the early stages of development, offspring have very high requirements for the quality and quantity of nutrients they need. Therefore, many adult animals use specific diets to meet the dietary requirements of their offspring (Brodmann and Reyer, 1999). The second reason may be due to the retention and digestion time in the intestines of birds (McWhorter et al., 2009). Some types of food are digested so thoroughly that they cannot be successfully identified in feces. It is worth noting that, in addition to insects, some other animal species were also identified in the food of Beijing swift. It is speculated that this may be due to the mosquitoes that Beijing swift prey on having sucked the blood of other animals. Differences between fecal analysis and regurgitated pellets analysis in terms of food digestibility may lead to underestimation or overestimation of certain specific groups. Fecal analysis reflects the average diet during a specific period, while regurgitated pellets analysis typically only reflects the situation over a few hours or days (Cucco et al., 1993). Differences in the dietary habits of individual Beijing swift may be due to the spatio-temporal uncertainty of arthropod resources. The gregarious behavior of some insects can cause local prey to accumulate in large numbers, and opportunistic utilization of these dynamically changing resources can lead to extremely high intraspecific dietary variability.

As in all animals, diet exerts a profound influence on the avian microbiome composition (Waite and Taylor, 2014; Li et al., 2021). Consistent with other aerial insectivorous birds (Uehling and Houtz, 2025), the gut microbiota of Beijing Swifts is predominated by Proteobacteria and Firmicutes, which facilitate the degradation of proteins and lipids abundant in their insect-based diet (Borrelli et al., 2017). This profile acutely contrasts with that of herbivorous birds, which typically harbor greater proportions of Bacteroidota to facilitate degradation of complex plant polysaccharides such as cellulose (Matheen et al., 2022), whereas omnivorous birds feeding on fruits, nectar, and insects show a predominance of Tenericutes and Proteobacteria in their gut microbial communities (Bodawatta et al., 2018). In this study, the Firmicutes in the gut of Beijing Swifts may have played a crucial role in the metabolism, digestion, and absorption of proteins and other nutrients (Berry, 2016). Short-chain fatty acids produced by Firmicutes can be directly absorbed by the intestinal wall, providing energy for the host. The abundance of Firmicutes is associated with weight gain in humans, chickens, and rodents, but its role in wild birds remains unclear (Sun et al., 2023). Migratory birds face physiological challenges related to energy and immunity during migration, so they have evolved various physiological traits adapted to their migratory habits. The high abundance of the Firmicutes in the intestine of Beijing swift may help them obtain the energy needed during long distance migration. In this study, no significant differences were observed in either alpha or beta diversity between the intestinal microbiota of banded swifts from the Summer Palace and rehabilitated swifts from the rescue center, indicating that the gut microbiome of Beijing Swifts exhibits remarkable resilience or stability and may not be substantially altered by short-term rehabilitation and dietary changes.

Insectivorous birds primarily consume protein-rich arthropods. Genera such as *Enterococcus* and *Lactobacillus* dominate the digestive tracts of these animals. In the human digestive tract, one of their primary functions is carbohydrate metabolism (Hammes and Hertel, 2006; Pikuta, 2014), and it is expected that they will perform similar functions in the digestive tracts of birds. Many *Lactobacillus* species also produce antimicrobial substances, which may play a role in defensive antagonism. They may also contribute to detoxification by hydrolysing bile acids and removing by-products of protein hydrolysis (Pikuta, 2014). *Enterococcus* can hydrolyse amino acids, a process that has been shown to be important for energy production in carnivorous mammals (Leblanc, 2006). Therefore, it is conceivable that insectivorous birds with a protein-rich diet may benefit similarly from beneficial bacteria such as *Lactobacillus* by improving energy absorption and reducing harmful by-products of protein hydrolysis. This study also found that *Escherichia_Shigella*, a potential pathogen, was relatively abundant in the intestines of Beijing swift. Other studies have shown that this bacterial genus is also prevalent in the intestines of migratory birds during autumn migration (Ouyang et al., 2025). This is due to seasonal changes in the intestinal microbiota of migratory birds, influenced by dietary changes, environmental factors and physiological stress during migration. The higher prevalence of pathogens during autumn migration underscores the potential health risks for Beijing swifts and highlights the necessity of implementing targeted protective strategies at stopover sites. Birds can carry many emerging infectious diseases and zoonotic pathogens, often harboring pathogenic microorganisms externally (Grond et al., 2018). In this study, low abundance *Salmonella*, a common genus of avian pathogens, was detected in the intestines of B14 and C1. These findings further our understanding of the dynamic interactions between wild bird migration and intestinal microbiota, providing valuable insights for ecological and health management.

The correlation network analysis indicated that genera including *Stenotrophomonas*, *Aminobacter*, *Ochrobactrum*, *Rhodococcus* and *Pseudomonas* constitute key taxa in the intestine of the Beijing Swift. These key bacteria likely form a highly synergistic functional group dedicated to xenobiotic degradation and detoxification within the swift’s gut. They are presumably capable of cooperatively degrading complex organic compounds, recalcitrant substrates, and specific contaminants such as pesticides present in the insect-based diet (Ryan et al., 2009; Artuso et al., 2021; Jaffar et al., 2022). As an aerial insectivore, the Beijing Swift preys on flying insects that may accumulate both plant secondary metabolites and synthetic environmental contaminants. Thus, the presence of these key bacterial groups may constitute an essential microbial defense mechanism, enabling the swifts to adapt to a high-protein diet with potential chemical risks, and playing a critical role in maintaining gut homeostasis and environmental adaptability during migration.

## 5 Conclusion

This study investigates the dietary composition and intestine microbiome of the Beijing Swift through the analysis of regurgitated pellets and fecal samples. Our findings reveal significant dietary differentiation between adults and nestlings. Core bacterial genera in adult swifts, such as *Stenotrophomonas* and *Aminobacter*, exhibit synergistic interactions with other intestinal microbes, suggesting functional cooperation within the microbial community. Collectively, this research establishes a scientific basis for future efforts to customize diets and evaluate the health of rescued swifts. However, this study has several limitations. First, sampling was restricted to the breeding season, preventing insights into dietary habits and gut microbiota dynamics of Beijing swifts during migration and at wintering grounds. Second, while amplicon sequencing effectively characterized microbial and dietary community composition, it cannot directly reveal functional profiles. Therefore, future research will pursue two main directions: temporally, by integrating satellite telemetry or stable isotope analysis with sample collection from banded swifts along migratory routes and wintering areas to fully resolve dietary patterns across their annual cycle; and technically, by employing multi-omics approaches such as metagenomics and metatranscriptomics to reconstruct complete metabolic pathways of key microbial taxa identified here, directly testing their hypothesized roles in xenobiotic degradation and detoxification, thereby fully elucidating the co-evolutionary adaptations between Beijing swifts and the environment.

## Data Availability

The datasets presented in this study can be found in online repositories. The names of the repository/repositories and accession number(s) can be found at: https://www.ncbi.nlm.nih.gov/, PRJNA1310792.

## References

[ref1] Akesson S. Klaassen R. Holmgren J. Fox J. W. Hedenström A. (2012). Migration routes and strategies in a highly aerial migrant, the common swift *Apus apus*, revealed by light-level geolocators. PLoS One 7:e41195. doi: 10.1371/journal.pone.0041195, PMID: 22815968 PMC3399846

[ref2] Artuso I. Turrini P. Pirolo M. Lugli G. A. Ventura M. Visca P. (2021). Phylogenomic reconstruction and metabolic potential of the genus *Aminobacter*. Microorganisms. 9:1332. doi: 10.3390/microorganisms9061332, PMID: 34205374 PMC8235418

[ref3] Berry D. (2016). The emerging view of Firmicutes as key fibre degraders in the human gut. Environ. Microbiol. 18, 2081–2083. doi: 10.1111/1462-2920.13225, PMID: 26842002

[ref4] Bodawatta K. H. Hird S. M. Grond K. Poulsen M. Jønsson K. A. (2022). Avian gut microbiomes taking flight. Trends Microbiol. 30, 268–280. doi: 10.1016/j.tim.2021.07.003, PMID: 34393028

[ref5] Bodawatta K. H. Sam K. Jønsson K. A. Poulsen M. (2018). Comparative analyses of the digestive tract microbiota of new guinean passerine birds. Front. Microbiol. 9:1830. doi: 10.3389/fmicb.2018.01830, PMID: 30147680 PMC6097311

[ref6] Borrelli L. Coretti L. Dipineto L. Bovera F. Menna F. Chiariotti L. . (2017). Insect-based diet, a promising nutritional source, modulates gut microbiota composition and SCFAs production in laying hens. Sci. Rep. 7:16269. doi: 10.1038/s41598-017-16560-6, PMID: 29176587 PMC5701250

[ref7] Brodmann P. A. Reyer H. U. (1999). Nestling provisioning in water pipits (*Anthus spinoletta*): do parents go for specific nutrients or profitable prey? Oecologia 120, 506–514. doi: 10.1007/s004420050884, PMID: 28308300

[ref8] Cucco M. Bryant D. M. Malacarne G. (1993). Differences in diet of common (*Apus apus*) and pallid (*A. pallidus*) swifts. Avocetta 17, 131–138.

[ref9] De Vos W. M. Tilg H. Van Hul M. Cani P. D. (2022). Gut microbiome and health: mechanistic insights. Gut 71, 1020–1032. doi: 10.1136/gutjnl-2021-326789, PMID: 35105664 PMC8995832

[ref10] del Hoyo J. Elliott A. Sargatal J. (1999). Handbook of the birds of the world, Vol. 5: Barn owls to hummingbirds. Barcelona: Lynx Edicions.

[ref11] Grond K. Sandercock B. K. Jumpponen A. Zeglin L. H. (2018). The avian gut microbiota: community, physiology and function in wild birds. J. Avian Biol. 49:e01788. doi: 10.1111/jav.01788

[ref12] Hammes W. P. Hertel C. (2006). “The genera of *Lactobacillus* and *Carnobacterium*” in The prokaryotes: A handbook on the biology of Bacteria. eds. Dworkin M. Falkow S. Rosenberg E. Schleifer K. Stackebrandt E. (New York, NY: Springer-Verlag), 320–403.

[ref13] Jaffar S. Ahmad S. Lu Y. (2022). Contribution of insect gut microbiota and their associated enzymes in insect physiology and biodegradation of pesticides. Front. Microbiol. 13:979383. doi: 10.3389/fmicb.2022.979383, PMID: 36187965 PMC9516005

[ref14] Kopij G. (2000). Diet of swifts (Apodidae) and swallows (Hirundinidae) during the breeding season in south African grassland. Acta Ornithol. 35, 203–206. doi: 10.3161/068.035.0201

[ref15] Leblanc D. J. (2006). “Enterococcus” in The prokaryotes (New York, NY: Springer), 175–204.

[ref16] Li C. Liu Y. Gong M. Zheng C. Zhang C. Li H. . (2021). Diet-induced microbiome shifts of sympatric overwintering birds. Appl. Microbiol. Biotechnol. 105, 5993–6005. doi: 10.1007/s00253-021-11448-y, PMID: 34272578

[ref17] Li L. X. Ye J. L. Yu M. C. Jiang J. X. Guo X. Y. Yu W. J. . (2025). Dynamic changes in the avian gut microbiome in response to diverse lifestyles. Ibis 167, 331–344. doi: 10.1111/ibi.13388

[ref18] Matheen M. I. A. Gillings M. R. Dudaniec R. Y. (2022). Dominant factors shaping the gut microbiota of wild birds. Emu 122, 255–268. doi: 10.1080/01584197.2022.2114088

[ref19] McWhorter T. J. Caviedes-Vidal E. Karasov W. H. (2009). The integration of digestion and osmoregulation in the avian gut. Biol. Rev. Camb. Philos. Soc. 84, 533–565. doi: 10.1111/j.1469-185X.2009.00086.x, PMID: 19673857

[ref20] Ouyang X. Q. Guan Y. Pei J. C. Ge J. P. Wang H. F. Bao L. (2025). Seasonal variation in gut microbiota of migratory wild raptors: a case study in white-tailed eagles. Anim. Microbiome 7:37. doi: 10.1186/s42523-025-00406-y, PMID: 40247417 PMC12007228

[ref21] Pikuta E. V. (2014) in Section 2: Family Carnobacteraceae in the lactic acid Bacteria: Biodiversity and taxonomy. eds. Holzapfel W. H. Wood B. J. B.. 1st ed (Hoboken, NJ: John Wiley & Sons, Ltd), 107–108.

[ref23] Ryan R. P. Monchy S. Cardinale M. Taghavi S. Crossman L. Avison M. B. . (2009). The versatility and adaptation of bacteria from the genus Stenotrophomonas. Nat. Rev. Microbiol. 7, 514–525. doi: 10.1038/nrmicro2163, PMID: 19528958

[ref24] Somers S. E. Davidson G. L. Johnson C. N. Reichert M. S. Crane J. M. S. Ross R. P. . (2023). Individual variation in the avian gut microbiota: the influence of host state and environmental heterogeneity. Mol. Ecol. 32, 3322–3339. doi: 10.1111/mec.16919, PMID: 36906957

[ref25] Sun F. F. Chen J. F. Liu K. Tang M. Z. Yang Y. W. (2022). The avian gut microbiota: diversity, influencing factors, and future directions. Front. Microbiol. 13:934272. doi: 10.3389/fmicb.2022.934272, PMID: 35992664 PMC9389168

[ref26] Sun Y. Zhang S. Nie Q. He H. Tan H. Geng F. . (2023). Gut firmicutes: relationship with dietary fiber and role in host homeostasis. Crit. Rev. Food Sci. Nutr. 63, 12073–12088. doi: 10.1080/10408398.2022.2098249, PMID: 35822206

[ref27] Uehling J. J. Houtz J. L. (2025). Gut microbiome–diet interactions in wild birds. J. Avian Biol. 2025:e03456. doi: 10.1002/jav.03456

[ref28] Waite D. W. Taylor M. W. (2014). Characterizing the avian gut microbiota: membership, driving influences, and potential function. Front. Microbiol. 5:223. doi: 10.3389/fmicb.2014.00223, PMID: 24904538 PMC4032936

[ref29] Wang X. T. (1985). Preliminary study on the ecology of the Beijing swift in Lanzhou. Biol. Bull. 7, 15–18.

[ref31] Youngblut N. D. Reischer G. H. Walters W. Schuster N. Walzer C. Stalder G. . (2019). Host diet and evolutionary history explain different aspects of gut microbiome diversity among vertebrate clades. Nat. Commun. 10:2200. doi: 10.1038/s41467-019-10191-3, PMID: 31097702 PMC6522487

[ref32] Zhang L. Tuoliken H. Li J. Gao H. L. (2025). Diet, gut microbiota, and health: a review. Food Sci. Biotechnol. 34, 2087–2099. doi: 10.1007/s10068-024-01759-x, PMID: 40351733 PMC12064509

[ref33] Zheng Z. X. (1993). Economic birds of China. Beijing: Science Press.

