# Supplementary material for: Revealing dietary habits and intestinal microbiome composition of the Beijing swift (Apus apus pekinensis) through regurgitated pellets and fecal samples
Source: Front Microbiol. 2025 Dec 1;16:1693396. doi: 10.3389/fmicb.2025.1693396 (PMC12702730; doi:10.3389/fmicb.2025.1693396)
Supplement: Supplementary file 1 [file Data_Sheet_1.docx]

Supplementary Material

# Supplementary Figures and Tables

## Supplementary Figures


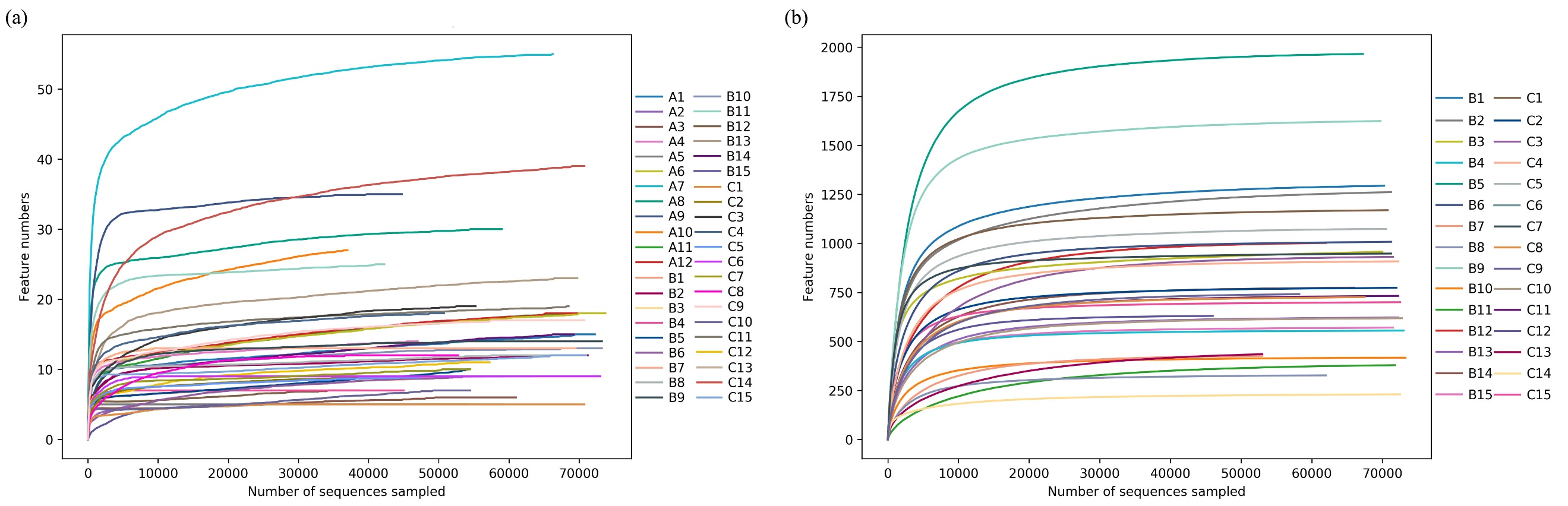


**Supplementary Figure 1.** Rarefaction curves. (a) Rarefaction curves for 16S (V3-V4) sequencing and (b) Rarefaction curves for COI gene sequencing. The x-axis shows the number of sequences per sample and the y-axis shows the feature numbers. Each curve in the graph represents a different sample and is shown in a different color.
